# Identification of the hypertension drug niflumic acid as a glycine receptor inhibitor

**DOI:** 10.1038/s41598-020-70983-2

**Published:** 2020-08-19

**Authors:** Daishi Ito, Yoshinori Kawazoe, Ayato Sato, Motonari Uesugi, Hiromi Hirata

**Affiliations:** 1 Department of Chemistry and Biological Science, College of Science and Engineering, Aoyama Gakuin University, Sagamihara, 252-5258 Japan; 2 Saga University Center for Education and Research in Agricultural Innovation, Karatsu, 847-0021 Japan; 3 Institute of Transformative Bio-Molecules (WPI-ITbM), Nagoya University, Nagoya, 464-8601 Japan; 4 Institute for Chemical Research (ICR) and Institute for Integrated Cell-Material Sciences (WPI-iCeMS), Kyoto University, Uji, 611-0011 Japan

**Keywords:** Developmental biology, Embryogenesis, Drug discovery, Drug screening, Phenotypic screening

## Abstract

Glycine is one of the major neurotransmitters in the brainstem and the spinal cord. Glycine binds to and activates glycine receptors (GlyRs), increasing Cl^−^ conductance at postsynaptic sites. This glycinergic synaptic transmission contributes to the generation of respiratory rhythm and motor patterns. Strychnine inhibits GlyR by binding to glycine-binding site, while picrotoxin blocks GlyR by binding to the channel pore. We have previously reported that bath application of strychnine to zebrafish embryos causes bilateral muscle contractions in response to tactile stimulation. To explore the drug-mediated inhibition of GlyRs, we screened a chemical library of ~ 1,000 approved drugs and pharmacologically active molecules by observing touch-evoked response of zebrafish embryos in the presence of drugs. We found that exposure of zebrafish embryos to nifedipine (an inhibitor of voltage-gated calcium channel) or niflumic acid (an inhibitor of cyclooxygenase 2) caused bilateral muscle contractions just like strychnine-treated embryos showed. We then assayed strychnine, picrotoxin, nifedipine, and niflumic acid for concentration-dependent inhibition of glycine-mediated currents of GlyRs in oocytes and calculated IC_50_s. The results indicate that all of them concentration-dependently inhibit GlyR in the order of strychnine > picrotoxin > nifedipine > niflumic acid.

## Introduction

Glycine, one of the major neurotransmitters, binds to glycine receptor (GlyR), mediating fast inhibitory synaptic transmission in the brain stem and the spinal cord^1,2^. Inhibitory glycinergic transmission is involved in generating rhythms such as respiration and walking/running. GlyRs are pentameric ligand-gated chloride-permeable channels. Extensive studies of mammalian GlyRs have identified four α subunit genes (*GLRA1, GLRA2, GLRA3* and *GLRA4*) and a single β subunit gene (*GLRB*) with the *GLRA4* being a pseudogene in human^3–8^. Since mutations in a gene encoding α1 or β subunit of GlyR causes startle reflex defects, which are often referred to as hyperekplexia in human, the major GlyRs in mammals is composed of α1 and β subunits^9,10^. GlyRs have also been studied in zebrafish, a vertebrate model, that offer several advantages such as production of many offspring, fast development, optical transparency during embryogenesis and ease of pharmacological assay. Zebrafish have five α subunit (*glra1, glra2, glra3, glra4a* and *glra4b*) and two β subunit (*glrba* and *glrbb*) genes^11^. The existence of two paralogs of a mammalian gene is not uncommon in zebrafish due to an ancestral gene duplication during fish evolution^12^. Both *glra1* mutant and *glrbb* mutant zebrafish showed touch-evoked simultaneous contractions of bilateral muscles, and as a consequence startle reflex just like strychnine-treated zebrafish embryos exhibited^13,14^. Thus, the major GlyRs in zebrafish embryos comprise α1 and βb subunits as in mammals.

All α subunits form homopentameric GlyRs activated by glycine and inhibited by strychnine and picrotoxin^15^. The β subunits, on the other hand, do not form homomers, while they are incorporated in heteropentameric αβ GlyRs, which is activated by glycine and inhibited by strychnine^5^. Regardless of homomeric α GlyRs or heteromeric αβ GlyRs, glycine binds to the extracellular intersubunit sites, where strychnine also binds as a competitive antagonist and blocks gating of the channel^16^. Picrotoxin binds to the second transmembrane domain of GlyR and clog the channel pore^17^. Interestingly, picrotoxin blocks homomeric α GlyRs at low concentration (~ 10 μM), while ten folds more picrotoxin is necessary to block heteromeric αβ GlyRs in mammals^18^. Collectively, these inhibitors provided striking insights to extend our understanding of GlyR properties. Identification and characterization of new GlyR inhibitors are expected to further improve our knowledge of GlyRs.

To search for new chemical compounds that block GlyRs, we screened a chemical library of ~ 1,000 approved drugs and pharmacologically active molecules through their ability to cause touch-evoked bilateral muscle contractions in zebrafish embryos. Strychnine served as a positive control. The screening identified nifedipine and niflumic acids as candidates of GlyR inhibitors. We also found that picrotoxin also affects zebrafish behavior when applied at high concentration. Our electrophysiological recordings using *Xenopus* oocytes revealed that all of the strychnine, picrotoxin, nifedipine and niflumic acids showed concentration-dependent blockade of glycine-gated currents in both homomeric and heteromeric GlyRs. In both human and zebrafish GlyR cases, the half-maximal inhibitory concentration (IC_50_) was strychnine < picrotoxin < nifedipine < niflumic acid.

## Results

### Bath application of nifedipine or niflumic acid affects tactile response of zebrafish

We have previously reported that zebrafish embryos swim when touched at 24 and 48 h postfertilization (hpf), whereas strychnine-treated embryos show obvious shrinkage of the body without swimming due to the loss of GlyR-mediated reciprocal inhibition^13,19^. We assumed that in vivo screening of chemical compounds that cause touch-evoked body shrinkage of zebrafish is a promising way to search for new GlyR inhibitors. To this end, three zebrafish embryos were treated with a drug (10 ~ 100 μM) in a well of 96-well plate at the onset of 24 hpf and then assayed for touch responses at 27 and 48 hpf, when zebrafish embryos show tactile-induced short-distance forward movement and escape swimming, respectively^20^. Among ~ 1,000 drugs that have some known physiological activities, about 79% of the drugs did not affect tactile response, while 10% caused immotile and 10% killed embryos at both stages in our first screening performed in a blind manner. We found 10 drugs that caused touch-evoked body shrinkage or weird uncoordinated response, the latter with contractions of small amplitude and/or compromised rhythmicity (Table 1). We then purchased these drugs, performed second round of screening at several different concentrations and identified three drugs that caused touch-evoked body shrinkage: strychnine, nifedipine and niflumic acid, which are known as specific GlyR inhibitor, voltage-gated calcium channel inhibitor and cyclooxygenase inhibitor, respectively. Successful identification of strychnine in our assay indicates that our screening works well. Nifedipine was previously suggested as a GlyR inhibitor^21^. Although picrotoxin, the other known GlyR inhibitor, was included in our chemical library, we failed to identify it in our screening.

**Table 1:** List of chemicals and touch-evoked responses of zebrafish embryos.

Chemical	Response 1st screening	Response 2nd screening	Known effect	Structure	MW
Strychnine	Body shrinkage	Body shrinkage	GlyR antagonist	C21H22N2O2	334
Nifedipine	Body shrinkage	Body shrinkage	Calcium channel blocker	C17H18N2O6	346
Niflumic acid	Weird contraction	Body shrinkage	Anti-inflammatory agent	C13H9F3N2O2	282
Mefenamic acid	Body shrinkage	Normal	Anti-inflammatory agent	C15H15NO2	241
Heptaminol hydrochloride	Body shrinkage	Normal	Myocardial stimulant	C8H20ClNO	182
Flavoxate hydrochloride	Weird contraction	Normal	Smooth muscle relaxant	C24H26ClNO4	428
Dicumarol	Weird contraction	Normal	Depletes vitamin K	C19H12O6	336
Antimycin A	Weird contraction	Normal	Antibiotics	C28H40N2O9	549
Papaverine hydrochloride	Weird contraction	Normal	Phosphodiesterase inhibitor	C20H22ClNO4	376
Nitrarine dihydrochloride	Weird contraction	Normal	Antiarrhythmic drug	C20H27Cl2N3	380

In the first screening, application of either of ten chemicals to zebrafish embryos caused touch-evoked body shrinkage or weird body contraction. The second screening identified thee chemicals as for further analysis.

**Figure 1:**
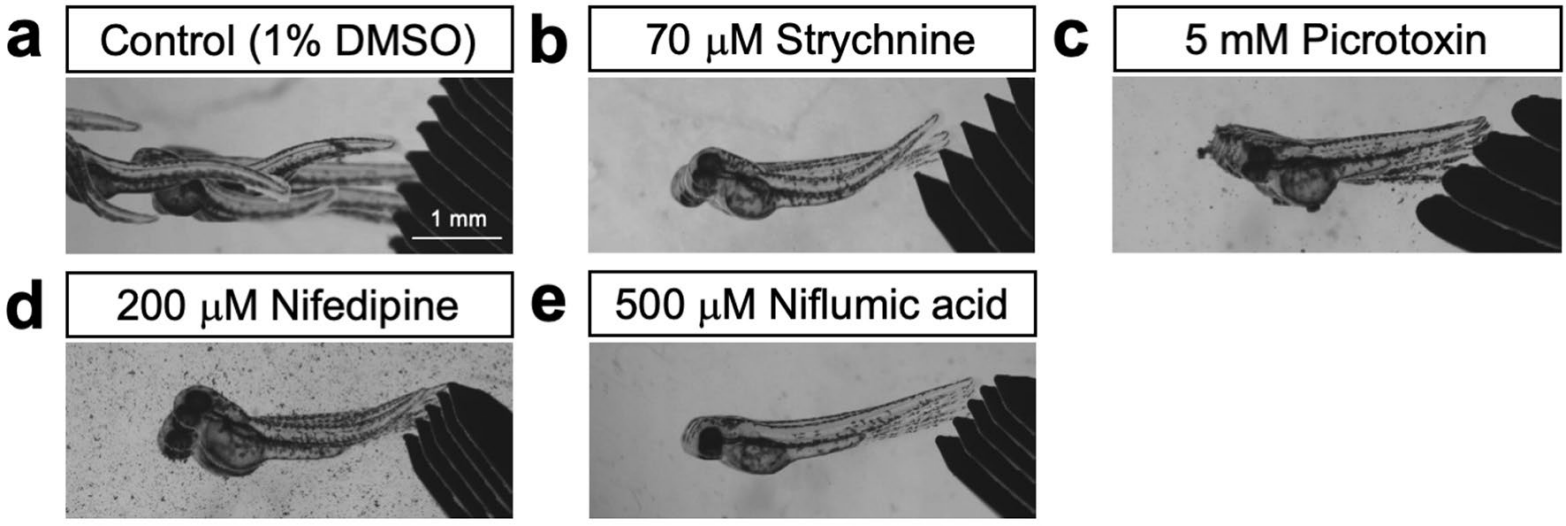
Bath application of strychnine, picrotoxin, nifedipine or niflumic acid to zebrafish embryos at 48 hpf caused abnormal touch response touch. (**a**) A superimposed image of 100 Hz movie frames. A control wild-type embryo (1% DMSO) responded to tactile stimulation and showed swimming by side-to-side trunk contractions. (**b**) Strychnine-treated embryos responded to touch by bilateral muscle contractions that resulted in shortening of the body along anterior–posterior axis. (**c**) Picrotoxin-treated embryos exhibited similar shrinkage of the body. (**d**) Nifedipine-treated embryos showed similar abnormal touch response. (**e**) Niflumic acid-treated embryos also responded to touch by bilateral muscle contractions.

We then detailed tactile response of zebrafish embryos at 48 hpf for four drugs: strychnine, picrotoxin, nifedipine and niflumic acid. Upon touch at the tail, control (1% DMSO) zebrafish embryos showed escape swimming by rhythmic side-to-side trunk contractions (Fig. 1a; Supplementary video 1). The shape of the notochord was straight during swimming. Embryos treated with 70 μM strychnine looked normal before touch. However, when touched, they initially exhibited bilateral trunk contractions resulting in the shrinkage of the body and eventually recovered to the normal state (Fig. 1b; Supplementary video 2). The shape of the notochord became zigzag due to the abnormal body compression along the anterior–posterior axis. Bath application of embryos with picrotoxin at 5 mM but not at lower concentrations caused shrinkage of the body following tactile stimulation. Their notochord was corrugated during abnormal touch response (Fig. 1c; Supplementary video 3). Embryos treated with nifedipine at 200 μM displayed bilateral muscle contractions, and as a consequence dorsally bent of the body just like strychnine-treated embryos showed (Fig. 1d; Supplementary video 4). The shape of the notochord was distorted during the response. Likewise, exposure of embryos with 500 μM niflumic acid induced touch-evoked shrinkage of the body and zigzag notochord (Fig. 1e; Supplementary video 5). These abnormal touch responses in the presence of either four drugs were indistinguishable from touch-evoked behaviors of zebrafish GlyR α1 and GlyRβb mutants^13,14^. These results indicate that our in vivo chemical screening using zebrafish embryos was sufficient to identify potential GlyR inhibitors. The order of drug concentration efficient for inducing abnormal touch response was strychnine (70 μM) < nifedipine (200 μM) < niflumic acid (500 μM) < picrotoxin (5 mM).

### Homomeric and heteromeric GlyRs of zebrafish and human origins were formed

**Figure 2:**
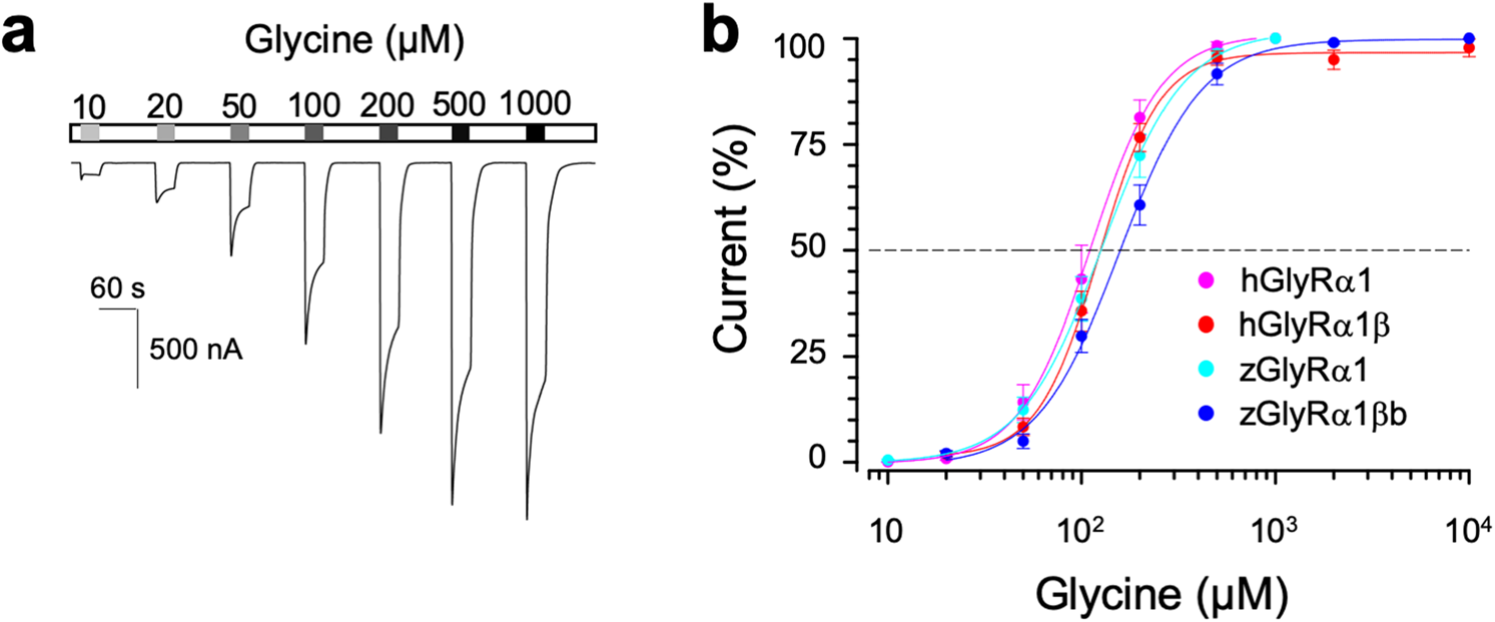
Heteromeric GlyR decreased glycine sensitivity compared with homomeric GlyR. (**a**) A trace of two-electrode voltage-clamp recording from oocyte injected with five femtomoles of zGlyRα1 cRNAs exposed to serial application of glycine of increasing amounts. (**b**) Cumulative concentration–response relationship of glycine-evoked currents. The amplitude of glycine-evoked currents was normalized to the maximally-evoked current as 100% for each oocyte (n = 10). Values here and elsewhere represent mean ± SEM.

To assess the drug sensitivity of GlyRs, we employed electrophysiology using *Xenopus* oocytes. We first checked glycine-mediated gating of α1 and β subunits of GlyR of zebrafish and human origins, as these subunits were the major GlyR components in vertebrates^9,10,13,14^. We injected cRNAs encoding zebrafish α1 subunit into oocytes, recorded glycine-dependent currents by two-electrode voltage-clamp and detected a concentration-dependent increase of currents for the calculation of half-maximally activated receptor (EC_50_) and hill coefficients (Fig. 2a,b). Expression of zebrafish α1 subunit with βb subunit also yielded glycine currents, and the cumulative concentration-curve was slightly right-shifted compared with the homomeric zebrafish α1 GlyR case. Similarly, injection of human α1 subunit only and α1 subunit with β subunit in oocytes generated functional homomeric and heteromeric GlyRs, respectively. These zebrafish and human GlyRs were activated by micromolar amounts of glycine and their EC_50_s were comparable to those in a previous study^22^ (Table 2). Injection of zebrafish βb subunit only or human β subunit only into oocytes did not yield currents (data not shown). These data confirm that zebrafish homomeric α1 and heteromeric α1βb GlyRs, as well as human homomeric α1 and heteromeric α1β GlyRs, were properly formed in oocytes.

**Table 2:** Summary of glycine-dependent gating of homomeric and heteromeric zGlyR and hGlyR.

	EC_50_ (µM)	Hill coefficient
zGlyR α1	112 ± 10	2.7 ± 0.1
zGlyR α1 + zGlyRβb	*163 ± 16	2.5 ± 0.2
hGlyR α1	107 ± 7.1	2.9 ± 0.1
hGlyR α1 + hGlyRβb	121 ± 10.2	3.2 ± 0.2

Values represent the mean ± SEM from 10 oocytes each. Where indicated *p < 0.05 reflects difference between homomeric and heteromeric GlyR.

### Niflumic acid blocked both homomeric and heteromeric zebrafish GlyRs by millimolar amounts

**Figure 3:**
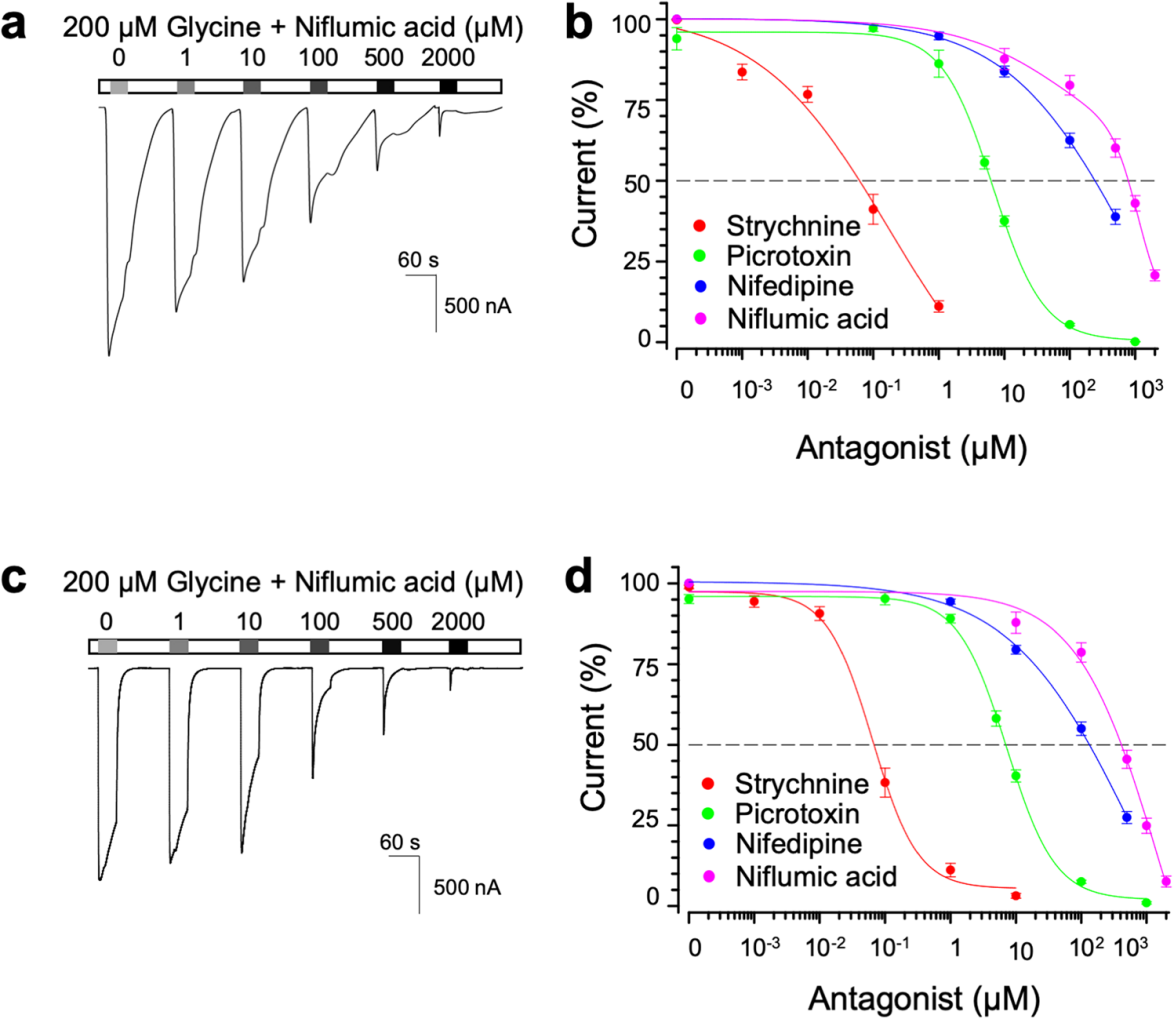
Drug-mediated inhibition of homomeric and heteromeric zebrafish GlyRs. (**a**) A recording from a zGlyRα1-injected oocyte exposed to 200 µM glycine with increasing amounts of niflumic acid. (**b**) Cumulative concentration-dependent inhibition of GlyRα1 current by strychnine, picrotoxin, nifedipine and niflumic acid (n = 10 each). (**c**) A recording from an oocyte, which were injected with zGlyRα1 and zGlyRβb and exposed to 200 µM glycine with increasing amounts of niflumic acid. (**d**) Cumulative concentration-curves of strychnine, picrotoxin, nifedipine and niflumic acid-mediated inhibition of α1/βb heteromeric zebrafish GlyRs (n = 10 each).

Since niflumic acid-treated zebrafish embryos as well as strychnine-, picrotoxin- and nifedipine-treated embryos exhibited abnormal touch response just like zebrafish GlyR mutants showed, niflumic acid likely affect GlyRs as strychnine, picrotoxin and nifedipine do. To address whether these drugs inhibit GlyR, we applied these drugs at different concentrations along with 200 μM glycine to *Xenopus* oocytes that express zebrafish GlyR α1, recorded currents and calculated IC_50_ and hill coefficients. In addition to strychnine, picrotoxin and nifedipine, niflumic acid showed concentration-dependent inhibition on homomeric zebrafish α1 GlyRs, suggesting that niflumic acid acts on homomeric α1 GlyRs as an inhibitor (Fig. 3a,b). Similarly, niflumic acid and the other three GlyR inhibitors caused concentration-dependent inhibition of heteromeric zebrafish α1βb GlyRs (Fig. 3c,d). The IC_50_s and hill coefficients of strychnine, picrotoxin and nifedipine were comparable between against homomeric α1 and against heteromeric α1βb GlyRs, while the IC_50_ of niflumic acid against homomeric α1 GlyRs were higher than that against heteromeric α1βb GlyRs, indicating that niflumic acid efficiently blocks heteromeric zebrafish GlyRs than homomeric zebrafish GlyRs (Table 3). Although the efficient drug concentrations that induce abnormal touch response in zebrafish embryos was strychnine < nifedipine < niflumic acid < picrotoxin, our electrophysiology suggested the order of IC_50_s as strychnine < picrotoxin < nifedipine < niflumic acid.

**Table 3:** Summary of drug-mediated inhibition properties of homomeric and heteromeric zGlyR and hGlyR.

	Strychnine		Picrotoxin		Nifedipine		Niflumic acid	
	IC_50_ [nM]	Hill coeff	IC_50_ [µM]	Hill coeff	IC_50_ [µM]	Hill coeff	IC_50_ [µM]	Hill coeff
zGlyR α1	64 ± 13	0.7 ± 0.2	6.1 ± 0.3	1.2 ± 0.1	239 ± 38	0.5 ± 0.1	801 ± 91	0.5 ± 0.1
zGlyR α1 + zGlyRβb	68 ± 12	0.9 ± 0.2	6.4 ± 0.3	0.9 ± 0.0	162 ± 24	0.4 ± 0.0	**426 ± 44	0.9 ± 0.1
hGlyR α1	72 ± 12	1.0 ± 0.1	7.2 ± 0.9	0.9 ± 0.1	171 ± 30	1.1 ± 0.2	1,074 ± 145	1.1 ± 0.2
hGlyR α1 + hGlyRβb	92 ± 19	0.8 ± 0.1	***94 ± 19	**0.5 ± 0.1	130 ± 27	0.9 ± 0.2	930 ± 111	1.1 ± 0.1

Values represent the mean ± SEM from 10 oocytes each. Where indicated **p < 0.01, ***p < 0.001 reflect difference between homomeric and heteromeric GlyRs.

### Niflumic acid blocked both homomeric and heteromeric human GlyRs at millimolar amounts

**Figure 4:**
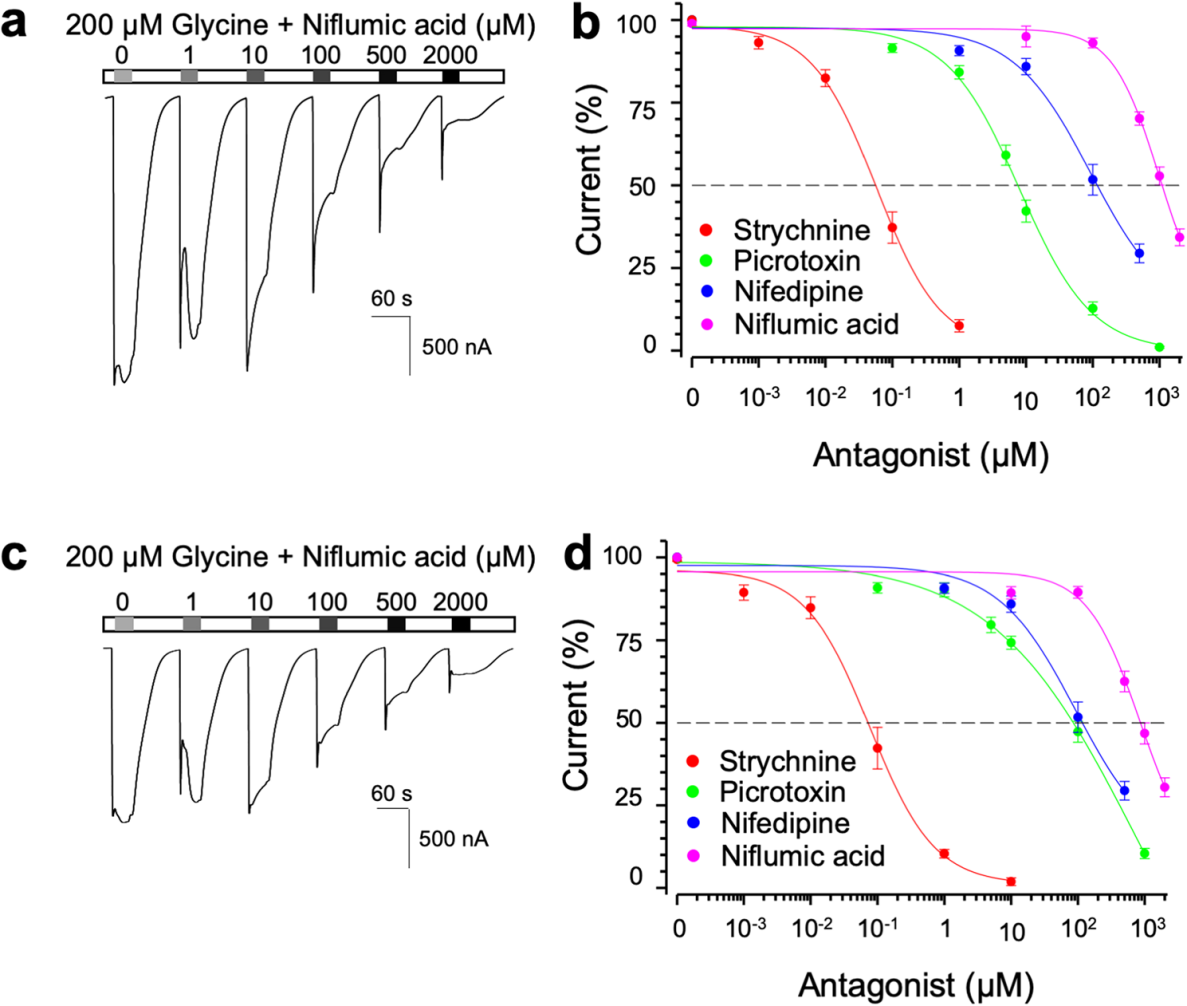
Drug-mediated inhibition of homomeric and heteromeric human GlyRs. (**a**) A recording from a hGlyRα1-injected oocyte exposed to 200 µM glycine with increasing amounts of niflumic acid. (**b**) Cumulative concentration-dependent inhibition of GlyRα1 current by strychnine, picrotoxin, nifedipine and niflumic acid (n = 10 each). (**c**) A recording from an oocyte, which were injected with hGlyRα1 and hGlyRβ and exposed to 200 µM glycine and increasing amounts of niflumic acid. (**d**) Cumulative concentration-curves of strychnine, picrotoxin, nifedipine and niflumic acid-mediated inhibition of α1/β heteromeric human GlyRs (n = 10 each).

We also investigated whether niflumic acid blocks human GlyRs. Exposure of niflumic acid at different concentrations with 200 μM glycine to *Xenopus* oocytes that express human GlyR α1 revealed that niflumic acid blocked GlyR in a concentration-dependent manner (Fig. 4a,b). Our electrophysiology confirmed that strychnine, picrotoxin and nifedipine also blocked homomeric human α1 GlyRs. Likewise, niflumic acid and the other three GlyR inhibitors showed concentration-dependent inhibition on heteromeric human α1β GlyRs (Fig. 4c,d). The IC_50_s and hill coefficients of strychnine, nifedipine and niflumic acid were comparable between against homomeric α1 and against heteromeric α1β GlyRs. As reported previously, the IC_50_ of picrotoxin against heteromeric human α1β GlyRs were significantly higher than that against homomeric human α1 GlyRs^18^. The order of IC_50_s was strychnine < picrotoxin < nifedipine < niflumic acid. Taken together, these results indicate that niflumic acid is an inhibitor of GlyR.

## Discussion

In this study, we employed chemical screening using zebrafish embryos to search for new GlyR inhibitors. Among ~ 1,000 approved drugs and pharmacologically active molecules, niflumic acid was identified as a candidate. We then performed *Xenopus* oocyte physiology and calculated IC_50_s and hill coefficients of niflumic acid against zebrafish and human GlyRs. Interestingly, the sufficient drug concentrations that induce abnormal touch response in zebrafish embryos were strychnine < nifedipine < niflumic acid < picrotoxin, the values of IC_50_ of GlyR was strychnine < picrotoxin < nifedipine < niflumic acid.

### In vivo chemical screening to identify new GlyR inhibitors

Although many zebrafish-based chemical screenings use transgenic or mutant zebrafish and thus need to select transgene-positive or mutation-carrying embryos/larvae, our screening uses wild-type zebrafish embryos at 48 hpf without any selection, enabling easy sample collection for the screening. In our chemical library, drugs were initially solved in DMSO and eventually diluted to 1/100 in each well of a 96-well plate for treatment. Thus, zebrafish embryos are exposed to drugs in the presence of 1% DMSO. This DMSO concentration facilitates the penetration of drugs into zebrafish body and is harmless. With these advantages, our in vivo drug screening tested ~ 50 drugs in a week and identified strychnine, nifedipine and niflumic acid as potential GlyR inhibitors. Interestingly, we failed to identify picrotoxin in this screening. But this was reasonable because 5 mM concentration of picrotoxin was necessary for inducing abnormal touch response. Since we succeeded in identifying three GlyR inhibitors from ~ 1,000 drugs, we will be able to find many novel ones if we expand the screening scale. While inhibition of GlyR impairs glycinergic transmission that results in the body shrinkage in zebrafish embryos, inhibition of glial glycine transporter 1 (GlyT1) causes synaptic accumulation of glycine and thus potentiates inhibitory glycinergic transmission, leading to reduction of touch response^23^. Although sarcosine, which is GlyT1 inhibitor, was not included in our chemical library, if it was, body contractions with small amplitude could be seen.

### Niflumic acid as a GlyR inhibitor

An anti-inflammation drug niflumic acid blocks cyclooxygenase-2 and is clinically used to reduce joint and muscular pain in rheumatoid^24^. It is also known as a blocker of calcium-activated chloride channels^25^, voltage-gated chloride channels^26^, NMDA receptors^27^ and GABA_A_ receptors^28^. Here we found that niflumic acid blocks both homomeric and heteromeric human GlyRs. Recent studies have investigated that niflumic acid affects human GlyRs expressed in CHO cells with higher blocking potency in homomeric α2 and α3 GlyRs than in homomeric α1 GlyRs^29^. Our electrophysiology results assayed in *Xenopus* oocytes were consistent with their human GlyR results. Since we could also reveal niflumic acid-sensitivity of zebrafish GlyRs and niflumic acid-induced motor deficits in zebrafish embryos, niflumic acid-mediated GlyR inhibition is assumed to be conserved among vertebrates.

### Effective drug concentration for blocking zebrafish GlyRs in *Xenopus* oocytes and zebrafish embryos

Our electrophysiology in *Xenopus* oocytes and touch-evoked response in zebrafish embryos revealed the effective concentration of drugs in vitro and in vivo, respectively. In the case of strychnine and picrotoxin, the effective concentrations for GlyR inhibition in zebrafish embryos (strychnine: 70 μM; picrotoxin: 5 mM) were 1,000 folds higher than the IC_50_ values of GlyRs expressed in *Xenopus* oocytes (strychnine: 70 nM; picrotoxin: 6 μM). This 1,000 fold difference is likely due to the low penetration of drugs through the skin. On the other hand for nifedipine and niflumic acid, the effective concentrations to induce abnormal touch response in zebrafish embryos (nifedipine: 200 μM; niflumic acid: 500 μM) were comparable to IC_50_s of GlyRs in oocyte physiology (nifedipine: 200 μM; niflumic acid: 400 μM). Since both of these drugs are insoluble to water and thus hydrophobic, they can be highly permeable to the skin.

## Material and methods

### Reagents

All chemicals and reagents except for library drugs for chemical screening were purchased from Wako Pure Chemical Industries and Thermo Fisher Scientific and used according to manufactures guidelines.

### Animal care and use

Zebrafish (*Danio rerio*) were reared and maintained at 28.5 °C under a 14 h light and 10 h dark photoperiod and fed twice a day in accordance with the Animal Care and Use Committee at Aoyama Gakuin University. Larvae were staged according to the established guidelines^30^, and are given as hours post-fertilization. At the ages we examined, sex determination has not yet occurred in zebrafish embryos.

### Drug screening

Two chemical libraries from Kyoto University and Nagoya University, composed of total ~ 1,000 bioactive compounds, were used in this study. The 2 µl of chemical compounds (1 ~ 10 mM) dissolved in DMSO were diluted to 200 µl with E3 medium (5 mM NaCl, 0.17 mM KCl, 0.33 mM CaCl_2_, 0.33 mM MgSO_4_) and transferred to 96-well plate. Three zebrafish embryos (24 hpf) were transferred into each well of the plate and incubated in a 28.5 °C incubator. At 27 hpf, three embryos with a small amount of drug solution were transferred to a 90-mm dish containing 15 ml of E3 medium and subjected to touch response using forceps under a dissection microscope Leica MZ16. After this assay, embryos with a small amount of E3 solution were transferred back to the drug solution in 96-well plate and incubated in the 28.5 °C incubator. At 48 hpf, embryos were transferred to a 90-mm dish containing 15 ml of E3 medium again and subjected to the touch response. The positive control for this assay was 70 µM strychnine, and the negative control was 1% DMSO.

### Video recording of zebrafish touch response

Tactile-evoked zebrafish movements in the presence of control 1% DMSO or drug were video recorded at 48 hpf using a dissection microscope. Touch responses elicited by tactile stimulation delivered to the tail with forceps were captured with a high-speed CCD camera at 200 frames per second (HAS-220, Ditect) as described previously^31^. Embryos were exposed to 70 µM strychnine, 5 mM picrotoxin, 200 µM nifedipine, 500 µM niflumic acid for 30 min before assay.

### Electrophysiology

Full-length cDNAs encoding zebrafish GlyR α1 and βb and human GlyR α1 and β subunits were used in this study. Capped cRNAs for expression in *Xenopus laevis* oocytes were synthesized from linearized templates using SP6 mMessage mMachine SP6 transcription kit (Thermo Fisher Scientific) as described previously^32^. Oocytes were injected with five femtomoles of cRNA using a Nanoject II (Drummond Scientific). Oocytes were moved to 48-well plates in Barth’s solution (88 mM NaCl, 1 mM KCl, 2.4 mM NaHCO_3_, 0.33 mM Ca (NO_3_)_2_, 0.41 mM CaCl_2_, 0.82 mM MgSO_4_, 10 mM HEPES at pH 7.5 with NaOH, supplemented with 50 µg/ml gentamicin and 100 units/ml penicillin/streptomycin) and incubated at 18 °C for 24–48 h prior to recording. Oocyte recording solution (90 mM NaCl, 1 mM KCl, 2 mM CaCl_2_, 1 mM MgCl_2_, 10 mM HEPES at pH 7.5 with NaOH) and drug solutions of seven different concentrations were flew into the oocyte chamber using a BPS-8 solution switcher (ALA Scientific). Borosilicate electrodes had resistances of ~ 2.0 MΩ when filed with 3 M KCl. Two-electrode voltage clamp recordings were made from oocytes held at − 70 mV using pClamp 10.2 to control a GeneClamp 500B amplifier via a Digidata 1440A digitizer (Molecular Devices) as described previously^33^. Signals were low-pass filtered at 10 Hz, and sampled at 100 Hz. Recordings were analyzed using Clampfit 10.7 (Axon Instruments) and SigmaPlot 11.0 (Systat Software, Inc.). EC_50_s, IC_50_s and Hill coefficients were calculated sigmoid standard curve as below.

$$\mathrm{y }=\mathrm{ min}+\frac{(\mathrm{max} - \mathrm{min})}{1+{(x/\text{EC}_{50})}^{-Hillslope}}$$

x: glycine concentration (EC_50_) or antagonist concentration (IC_50_). y: normalized current. Statistical significance was assessed using the pair-wise analysis of variance.

### Ethics statement

All animal experiments described in this manuscript and guidelines for use of zebrafish have been approved by Animal Care and Use Committee in Aoyama Gakuin University.

## Supplementary information

Supplementary file1

Supplementary file2

Supplementary file3

Supplementary file4

Supplementary file5

Supplementary file6
