# Supplementary material for: Identification of the hypertension drug niflumic acid as a glycine receptor inhibitor
Source: Sci Rep. 2020 Aug 19;10:13999. doi: 10.1038/s41598-020-70983-2 (PMC7438329; doi:10.1038/s41598-020-70983-2)
Supplement: Supplementary file 1 — Supplementary file1 [file 41598_2020_70983_MOESM1_ESM.pdf]

Identification of hypertension drug niflumic acid as a glycine receptor inhibitor

Daishi Ito, Yoshinori Kawazoe, Ayato Sato, Motonari Uesugi and Hiromi Hirata.

Supplementary videos

Supplementary video 1

Touch induced a zebrafish embryo (48 hpf) in control condition (1% DMSO) to swim away rapidly by side-to-side contractions of trunk muscles.

Supplementary video 2

A zebrafish embryo treated with 70 µM strychnine responded to touch by simultaneous bilateral muscle contractions that resulted in shortening of the body along anterior-posterior-axis.

Supplementary video 3

A zebrafish embryo treated with 5 mM picrotoxin showed shrinkage of the body following a tactile stimulation.

Supplementary video 4

A zebrafish embryo treated with 200 µM nifedipine exhibited dorsal bent of the body upon touch.

Supplementary video 5

A zebrafish embryo treated with 500 µM niflumic acid caused bilateral muscle contractions and as a consequence shortening of the body after touch.
